# Are the European reference networks for rare diseases ready to embrace machine learning? A mixed-methods study

**DOI:** 10.1186/s13023-024-03047-7

**Published:** 2024-01-25

**Authors:** Georgi Iskrov, Ralitsa Raycheva, Kostadin Kostadinov, Sandra Gillner, Carl Rudolf Blankart, Edith Sky Gross, Gulcin Gumus, Elena Mitova, Stefan Stefanov, Georgi Stefanov, Rumen Stefanov

**Affiliations:** 1 Institute for Rare Diseases, 22 Maestro G. Atanasov St., 4017 Plovdiv, Bulgaria; 2 Department of Social Medicine and Public Health, Faculty of Public Health, Medical University of Plovdiv, 15A Vasil Aprilov Blvd., 4002 Plovdiv, Bulgaria; 3 KPM Center for Public Management, University of Bern, Freiburgstr. 3, 3010 Bern, Switzerland; 4 Swiss Institute for Translational and Entrepreneurial Medicine (Sitem-Insel), Freiburgstr. 3, 3010 Bern, Switzerland; 5 EURORDIS – Rare Diseases Europe, 96 Rue Didot, 75014 Paris, France; 6 Department of Epidemiology and Disaster Medicine, Faculty of Public Health, Medical University, 15A Vasil Aprilov Blvd., 4002 Plovdiv, Bulgaria

**Keywords:** Rare diseases, Machine learning, Artificial intelligence, European reference networks, Diagnosis, Diagnostic delay

## Abstract

**Background:**

The delay in diagnosis for rare disease (RD) patients is often longer than for patients with common diseases. Machine learning (ML) technologies have the potential to speed up and increase the precision of diagnosis in this population group. We aim to explore the expectations and experiences of the members of the European Reference Networks (ERNs) for RDs with those technologies and their potential for application.

**Methods:**

We used a mixed-methods approach with an online survey followed by a focus group discussion. Our study targeted primarily medical professionals but also other individuals affiliated with any of the 24 ERNs.

**Results:**

The online survey yielded 423 responses from ERN members. Participants reported a limited degree of knowledge of and experience with ML technologies. They considered improved diagnostic accuracy the most important potential benefit, closely followed by the synthesis of clinical information, and indicated the lack of training in these new technologies, which hinders adoption and implementation in routine care. Most respondents supported the option that ML should be an optional but recommended part of the diagnostic process for RDs. Most ERN members saw the use of ML limited to specialised units only in the next 5 years, where those technologies should be funded by public sources. Focus group discussions concluded that the potential of ML technologies is substantial and confirmed that the technologies will have an important impact on healthcare and RDs in particular. As ML technologies are not the core competency of health care professionals, participants deemed a close collaboration with developers necessary to ensure that results are valid and reliable. However, based on our results, we call for more research to understand other stakeholders’ opinions and expectations, including the views of patient organisations.

**Conclusions:**

We found enthusiasm to implement and apply ML technologies, especially diagnostic tools in the field of RDs, despite the perceived lack of experience. Early dialogue and collaboration between health care professionals, developers, industry, policymakers, and patient associations seem to be crucial to building trust, improving performance, and ultimately increasing the willingness to accept diagnostics based on ML technologies.

## Background

In the EU, rare diseases (RDs) were first outlined as a health policy priority by the Community action programme on RDs (1999–2003). This programme defined RD as severe conditions affecting no more than 5 per 10,000 persons in the EU [1, 2]. Owing to their complex nature, RDs stood out as a distinctive domain for international coordinated action at the European and international levels [3, 4]. In this context, the EU adopted a series of legislative acts, culminating in the Council Recommendation of June 8, 2009, outlining further action in the RD field [3–6].

Even though each RD has a low prevalence, between 5000 and 8000 separate rare conditions are nowadays identified, affecting between 6 and 8% of the population at a certain point in their lives [2, 7]. Most RD patients actually have significantly less common diseases that could impact one in every 100 000 persons or less [2]. Because their numbers are very small on a national scale, these people are extremely isolated and vulnerable [2]. They often have a long delay in diagnosis, spending considerable time and resources in seeking advice and testing, commonly referred to as a diagnostic odyssey [8].

Machine learning (ML) represents a new paradigm in RD diagnostics and management. By examining massive volumes of phenotype and genotype data and discovering complex multiallelic patterns, ML-based tools have the potential to increase the precision and speed of RD diagnosis [9]. Nevertheless, the successful implementation of these new technologies encompasses three important milestones: availability (in terms of market authorization) [10]; accessibility (in terms of coverage or reimbursement) [10]; and routine application in clinical practice. Furthermore, compared to other health technologies, clinical studies on ML-based tools have limitations and often lack adapted, robust, and complete evidence, which results in vague and unreliable estimates of efficacy and cost effectiveness [11–13]. Finally, ML is far more than a diagnostic technique. It is a health system transformation modality that could produce significant changes and impacts at numerous layers [14].

While decisions on availability and accessibility are mainly made by regulators, payers, healthcare providers, and professionals, the routine application in clinical practice largely depends on the individual medical specialists’ knowledge, attitudes, and willingness to accept and adopt these new health technologies [15–17].

The European Reference Networks (ERNs) represent key opinion leaders in the field of RDs. These are virtual networks that connect healthcare providers across Europe. ERNs aim to facilitate discussion on complex or rare conditions that require highly specialised knowledge and concentrated expertise [18]. In 2017, the first 24 ERNs were established, incorporating over 900 highly specialised healthcare units from over 300 hospitals throughout EU Member States [18]. These were gradually joined by more than 600 new member centres of expertise, bringing the total number of ERN members to about 1500 by January 2022 [19].

Cooperation and transfer of knowledge among ERNs have proven to be a very efficient strategy to address RDs in Europe. The added value of ERNs to society is particularly high due to the rarity of these conditions, which implies both a limited number of patients and a scarcity of expertise within a single jurisdiction [2, 18, 20, 21]. ERNs thus unite the most crucial RD healthcare providers in the EU and play an important role in RD policymaking both at the EU and national levels.

The purpose of this study is to explore ERN members’ expectations towards and acceptance of ML and its potential application, to understand the key benefits and risks perceived regarding its potential use, and to identify the key factors being considered by ERN members to promote the use of and access to ML technologies in the diagnostic process.

## Methods

We applied a two-stage research framework consisting of an online survey followed by a focus group discussion. As there is no universally accepted definition of ML, prior to the survey design, we consulted with ML stakeholders and came to the following working definition: "Machine learning (ML) is a computer-aided technique that may help physicians make a diagnosis by using information from past patient data". This formulation was presented to all study participants during all stages of the research.

### Study setting

This study was conducted as part of the Screen4Care public–private partnership. Screen4Care is funded by the Innovative Medicines Initiative and aims to accelerate RD diagnosis through ML technologies and genetic newborn screening [22]. Thus, the project contributes to people living with a rare genetic disorder by reducing the delay in diagnosis, to a sustainable healthcare system by avoiding inconclusive consultations and costly misdiagnosis, as well as to effective treatments and efficient use of healthcare resources [22, 23].

Our study is positioned within Work Package 1 of the Scree4Care project. This work package aims to understand the business, ethical, and regulatory environment for RD screening and diagnosis in Europe [22]. In particular, Work Package 1 explores the complex decision-making process for funding, reimbursement, and adoption of health technologies based on genetic screening or ML. This is of paramount importance, as the Screen4Care project deliverables, once available, could be implemented in practice [22].

### Study participants

Our study targeted primarily medical professionals but also other individuals affiliated with any of the 24 ERNs. We therefore formed a convenience sample of all the health care professionals with publicly available email addresses who were listed either on the ERNs’ websites or on the Orphanet database.

Screening those websites and databases resulted in a total of 2212 individuals that we contacted by email. The recipients received an invitation to participate in the survey with an invitation letter that described the study. In addition, we approached ERN coordinators and asked them to share the survey link within their ERN. We did not provide any incentives for participation.

### Survey

We developed the scope and format of the survey based on a literature review on perceptions and expectations of clinical artificial intelligence applications, as identified by Scott et al. [24]. The questionnaire consisted of 23 questions grouped into four sections: (1) socio-demographic and career profile; (2) knowledge and attitude towards ML; (3) attitudes towards ML’s potential implementation and integration in healthcare; and (4) attitudes towards ML’s prospects for disease diagnosis. Each question contained a free text field for providing additional input.

The questionnaire was piloted among a small group of medical professionals to improve consistency and clarity. The full final survey is presented in Table 1 (Appendix). We started the survey on April 19, 2022, and sent monthly reminders using LimeSurvey. The survey was active until September 1, 2022.

### Focus group

We drew focus group participants from the respondents who declared their willingness to participate and provided their contact information in the survey. In total, we invited 42 individuals, and 10 finally confirmed their participation.

The focus group discussion took place online on October 19, 2022. We provided the participants with a list of eight questions and obtained their informed consent for audio-visual recording and transcription before the meeting. During the discussion, we reminded the attendees to take their own perspective and experience into account when advising on ML in the diagnostic process of RDs.

### Data analysis

Descriptive statistics were applied. The self-reported ML experience was used as the main factor for comparison and analysis of ML attitudes and expectations. Chi-square test and Mann–Whitney U-test were used to compare the group of respondents that reported no ML experience at all to those reporting limited or extensive experience. Statistical significance was considered if the *p*-value was less than 0.05.

### Ethics committee approval

Approval by an Ethics committee was not required for this research. The survey and the focus group discussion were sociological from a methodological point of view and did not involve clinical research.

## Results

### Survey results

#### Socio-demographic and career profile of the respondents

423 individual responses were collected. The highest number of responses came from ERN members based in Italy (n = 96, 22.7%), followed by those from Germany and the Netherlands (both n = 38, 9%) Table 2 (Appendix). Respondents indicated an average of 22.7 (SD = 11.3) years of professional experience. The three most common medical specialties were paediatrics (n = 128), nephrology (n = 102), and endocrinology (n = 57).

Medical professionals from ERKNet, the ERN on kidney diseases, most actively took part in the survey (129 respondents), followed by members of Endo-ERN, the ERN on endocrine conditions (72 respondents) Table 2 (Appendix). Only three ERNs returned less than 10 responses: the ERN on rare multisystemic vascular diseases VASCERN (n = 8), the ERN on neuromuscular diseases EURO-NMD (n = 6), and the ERN on connective tissue and musculoskeletal diseases ReCONNET (n = 4). Note that respondents could indicate their affiliation with multiple ERNs.

### Knowledge and attitude towards ML

**Fig. 1:**
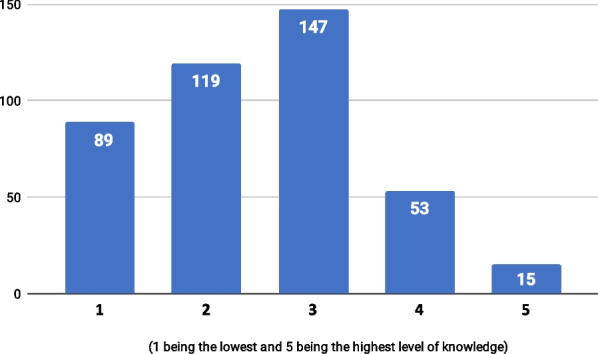
Distribution of respondents by self-assessed knowledge of ML on a 1–5 scale

**Fig. 2:**
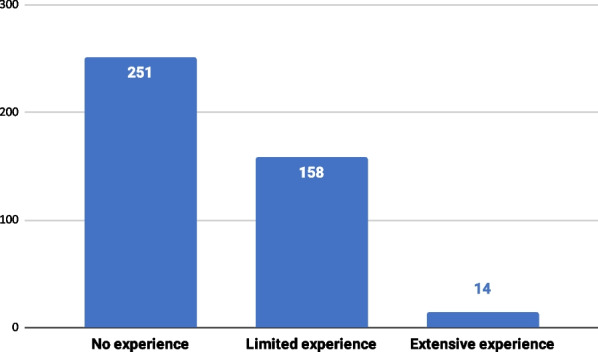
Distribution of respondents by self-reported ML experience

Respondents reported a relatively limited degree of knowledge of and experience with ML. About 50% (n = 208) assessed their own knowledge of ML on the lowest end of a 1–5 scale, whereas only 15 participants rated their knowledge at a 5 (Fig. 1). Similarly, around 60% (n = 251) declared to have never used ML in their clinical practice, and only 14 respondents indicated to have extensive ML experience (Fig. 2).

**Fig. 3:**
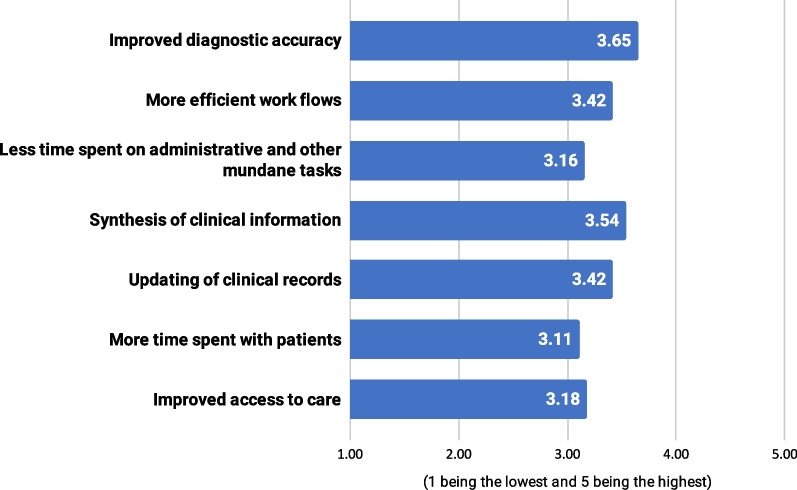
Assessment of ML potential benefits on a 1–5 scale

Survey participants assessed the importance of ML’s potential benefits and risks on a 1–5 scale, with 5 being the highest level. They rated improved diagnostic accuracy (mean = 3.65, SD = 1.52) as the most important benefit of ML, closely followed by synthesis of clinical information (mean = 3.54, SD = 1.47) (Fig. 3). Respondents who indicated having some ML experience consistently rated the benefits of ML higher. Improved diagnostic accuracy (4.09 vs. 3.35, *p* < 0.001), more efficient workflows (3.74 vs. 3.20, *p* = 0.015), and improved access to care (3.64 vs. 2.87, *p* < 0.001) were all graded significantly higher by this group compared to the group of participants who declared no ML experience.

**Fig. 4:**
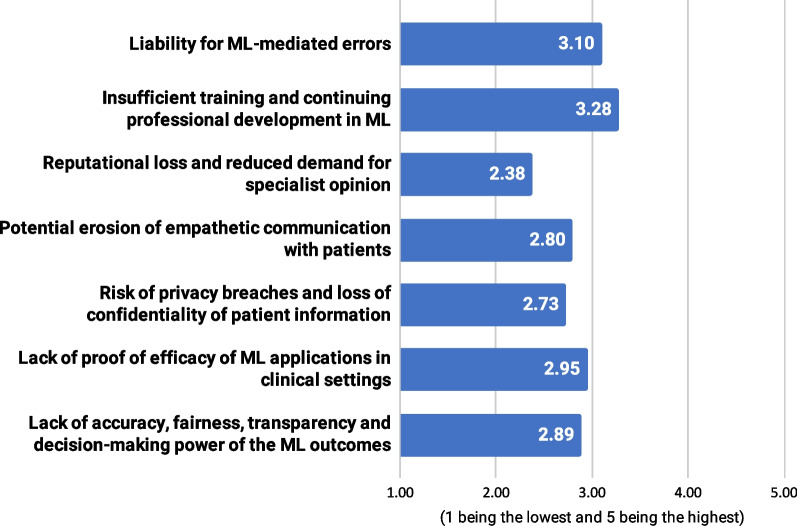
Assessment of ML potential risks on a 1–5 scale

The respondents identified insufficient training and continuing professional development around the use of ML for clinical purposes (mean = 3.28, SD = 1.50) as the most important risks of ML’s adoption and implementation, followed by liability for ML-mediated errors (mean = 3.10, SD = 1.57) (Fig. 4). They perceived reputational loss and reduced demand for specialist opinion (mean = 2.38, SD = 1.40) as the least important potential risks. Again, participants who reported some ML experience assessed the importance of those risks higher. Liability of ML-mediated errors (3.41 vs. 2.88, *p* = 0.047), insufficient training and continuing professional development (3.64 vs. 3.04, *p* = 0.002), and lack of accuracy, fairness, transparency, and decision-making power of the ML outcomes (3.22 vs. 2.67, *p* = 0.016) were all graded significantly higher compared to the group of participants who declared no ML experience.

### Attitudes towards ML’s potential implementation and integration in healthcare

Survey participants were asked to indicate their attitudes towards different modalities for ML’s potential implementation and integration in healthcare. Most respondents (n = 343, 81.1%) supported the option that ML should be an optional but recommended part of the diagnostic process for RDs. Only 33 respondents (7.8%) believed that ML technologies should be mandatory for RD diagnosis. The presence or lack of ML experience did have an impact on these findings (*p* = 0.034). A higher proportion of the participants who declared ML experience supported ML being a mandatory part (12.2% vs. 4.8%). On the other hand, 84.9% of the respondents without ML experience believed ML should be optional in this process, compared to 75.6% among those with ML experience.

**Fig. 5:**
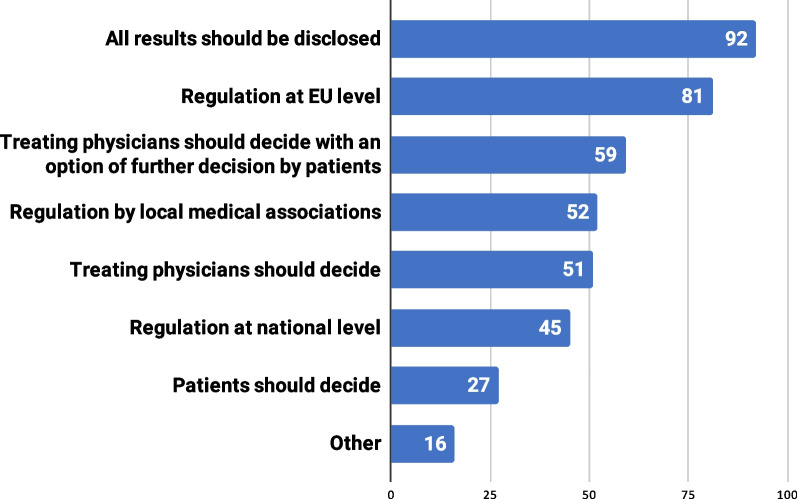
Distribution of respondents by preferred scope and type of ML diagnostic findings to be disclosed to patients

We could not find a clear consensus on the scope and type of ML diagnostic findings to be disclosed to RD patients (Fig. 5). While 22.7% (n = 92) believed that all ML diagnostic results should be disclosed to patients, 19.1% (n = 81) stated that there is a need for guidance at the EU level on what specific information to disclose.

**Fig. 6:**
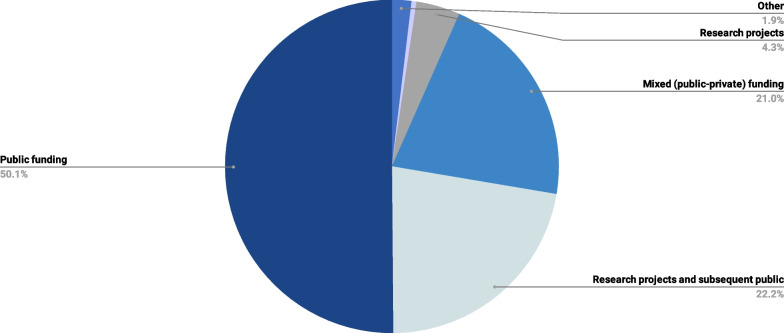
Distribution of respondents by preferred funding of ML diagnostics

Public funding was preferred as the main source to cover ML diagnostics of RDs by 50.1% (n = 212) of the survey’s participants (Fig. 6). The rest split their answers mainly between two options: funding through research projects and subsequent public funding if justified (n = 94, 22.2%), and mixed coverage (public–private funding) (n = 89, 21.0%). No significant association was found between the funding preferences and the self-reported ML experience (*p* = 0.144).

**Fig. 7:**
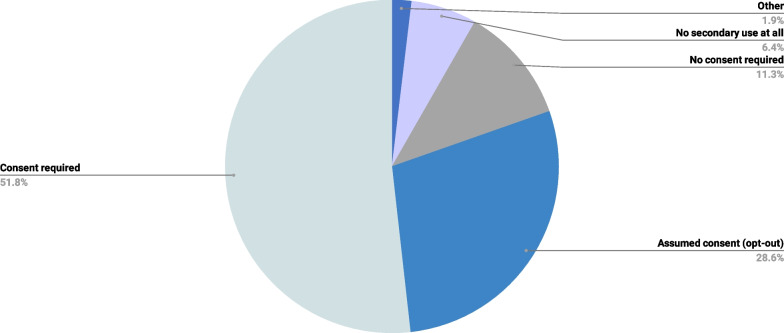
Distribution of respondents by preferred secondary use of ML anonymous data for research

About half of the respondents (n = 219, 51.8%) supported the notion that anonymized ML-generated diagnostic data should be available for secondary use only with patients’ consent (consent required), while 28.6% (n = 121) were open to sharing anonymized ML-generated diagnostic data for secondary research if patients did not opt-out explicitly (Fig. 7). We found no significant influence of the ML experience on these findings (*p* = 0.451).

### Attitudes towards ML’s prospects

A majority of respondents (n = 251, 59.3%) expected ML’s clinical application in the next 5 years to be restricted to specialised units (e.g., designated centres of expertise and ERNs). About a quarter (n = 109, 25.8%) expected no change from the current situation, and only 14.9% (n = 63) believed ML could be more widely applied in all clinical settings and all levels of health care, including autonomous application by patients. We found a significant association between the self-reported ML experience and the participants’ attitudes towards the future of ML (*p* < 0.001). Respondents with ML experience were more optimistic, with 20.3% believing that ML would be routinely applied in all clinical settings, compared to 11.2% among those with no ML experience. On the other hand, a higher portion of the individuals without ML experience expected no change from the current situation, 31.9% vs. 16.9%.

Survey participants were further asked to assess the importance of factors that could encourage the routine application of ML outside of research projects on a 1–5 scale, with 5 being the highest level of importance. Improving clinical decision-making and outcomes (mean = 4.06, SD = 1.30), ensuring accuracy, freedom from bias, and trustworthiness (mean = 3.96, SD = 1.42), and ensuring data privacy, confidentiality, and security (mean = 3.84, SD = 1.40) were considered most important for the routine application of ML. The respondents with and without ML experience only differed significantly regarding the importance of ensuring accuracy, freedom from bias, and trustworthiness, with the experienced group rating this factor higher (4.22 vs. 3.78, *p* = 0.029).

Respondents assessed the importance of several policy measures suggested to promote the routine application of and access to ML outside research projects on a 1–5 scale, with 5 being the most important. Among those, participants expressed the strongest support for focusing on improved effectiveness in clinical decision-making (mean = 3.84, SD = 1.35), and adherence to legal and community expectations regarding privacy, confidentiality, and security of health and medical data (mean = 3.82, SD = 1.45). Elaboration of regulatory standards that are robust, transparent, and responsive (mean = 3.74, SD = 1.37), clear lines of responsibility regarding liability for error (mean = 3.74, SD = 1.41), and respect for human-to-human interaction and shared decision-making (mean = 3.73, SD = 1.43) were highly appraised. Significant differences were found between the respondents with and without ML experience regarding the requirements for ML tools to be based on models that have involved domain experts (4.04 vs. 3.50, *p* = 0.006), to fit to and complement routine clinical workflows (3.60 vs. 3.13, *p* = 0.007), and to be developed with a focus on maximising explainability and transparency in terms of their inner workings (3.70 vs. 3.14, *p* = 0.002).

### Focus group results

Out of the 10 respondents initially confirming their participation in the online focus group, four participants attended online, and two participants provided their written responses instead. The participants were from six European countries and affiliated with seven ERNs.

The focus group lasted for 90 min. Two co-moderators provided discussion prompts following the structure of the online survey. Participants were presented with the survey’s preliminary outcomes before they were invited to comment with their own opinion and explore the answer patterns, while interaction between the focus group participants was encouraged by the co-moderators.

### Experience with ML in clinical practice

Focus group participants indicated having little to no experience with ML in their own clinical practice. Previous ML interactions came entirely from research projects. Nevertheless, there was a clear agreement that ML’s potential is substantial and will have an important impact on health care and the RD field.

Participant#1: *“I am not [using] it in my clinical practice myself, but I think it is a very interesting way of working. So, I feel that it's necessary to know more.”*

Participant#4: *“We have a project where we test ML for electronic records, … [but] it is not part of everyday clinical management yet. It is a pilot, and it aims to be an everyday technology when we know that it functions.”*

### Insufficient training as a main obstacle to the successful implementation of ML in the diagnostic process of RDs

Participants agreed that it is not the responsibility of medical specialists to be experts in ML. Nevertheless, they believed it necessary for clinicians to partner with ML tool developers to ensure that ML-generated outcomes are valid and reliable. To this end, ML training for physicians is considered beneficial if such training is tailored to the actual characteristics and needs of the various medical specialties and conditions. Some of the attendees favoured a top-down approach in the promotion of ML training, starting from highly specialised centres of expertise and eventually trickling down to undergraduate training.

Participant#1: *“It is necessary to have some work by medical societies – national, European, and international. They should promote ML in specific areas of interest and continuous education. And after that, we can think about promoting ML at the undergraduate level of training.”*

Participant#2: *“What is important for clinicians is to understand the process behind it. Use of ML commercial solutions does not require too much explanation and training, but it may require some training to better understand the technologies. I mean, in cases that are more research- and development-oriented, it is important to understand what is behind [the outcomes]. For example, whether, from a clinical perspective, the right data sets are used in the right way. Questions posed in this way make outcomes effective and reliable. However, the bulk of technical work behind it is not what physicians would need to understand or to work on. This is clearly the task of computer scientists and IT professionals.”*

Participant#4: *“I think we, as clinicians, do not need to be experts on the process behind the ML algorithm, but we need to be sure that the algorithm itself is valid and gives results that we can rely on. This is what is important. We do not need any training per se to develop these ML tools. We need, of course, collaboration with ML experts who know what ML can give us.”*

### Scope and type of ML diagnostic findings to be disclosed to RD patients

Most participants believe that current regulations and guidelines on the scope and type of diagnostic findings to be disclosed to patients are sufficient to respond well to ML’s implementation and its potential challenges.

Participant#2: *“We will decide based on the legislation and the clinical need. ML will be there only to increase our capacity. I do not see any difference if you get this type of information by ML or by any other technology.”*

Participant#4: *“ML is just a tool, and we already have regulations and guidelines for what kind of incidental findings are mandatory to disclose to the patient.”*

### Funding of ML diagnostics

Similar to the survey’s findings, participants stated that public funding should be the preferred option in case of RD diagnosis. Nevertheless, there must be a distinction between routine ML diagnostics in clinical settings and research projects. The latter should be funded by the current mix of public, private, and mixed resources.

*Participant#4: “When we talk about diagnostics, then it must, of course, be public funding because it is a tool for diagnostics and for the patients’ best [interest].”*

*Participant#5: “ML should be publicly funded, so that all patients can access it. Otherwise, it may become an add-on that only the wealthy or those with particular insurance can access.”*

*Participant#6: “If the evidence is sound and demonstrates dramatic improvement in RD diagnosis, the use of these models should be publicly funded and available, just like for new drugs or devices.”*

### ML application in the next five years

Participants mostly agreed that ML’s application in the next five years should be restricted to specialised units only. They stressed the fact that diagnosis also includes interpretation of the findings and communication with patients and their families.

*Participant#1: “…When we are dealing with RDs, it is not only the diagnosis, but it is also the interpretation and the information to the patients and their families. ML seems to be a very fast-moving field, but I do not see it [being applied broadly] right now. But who knows for the next five years.”*

*Participant#5: “It depends on what the ML is being used for. In a limited sense, various web programmes might be considered ML. Although "Dr. Google" has diagnosed some diseases, it has not been helpful in most cases, and patients become very anxious. However, in the appropriate hands, ML helps to identify a disease that clinicians may only see once in a lifetime and enables rapid referrals. I am in favour of ML for RDs being available locally but with support from centres of expertise. Local centres could use it but need training and must know who their specialty links are.”*

## Discussion

Our study aimed to explore whether members of ERNs are willing to use ML-based tools for RD diagnostics. However, ultimate decisions on the adoption of and access to these technologies depend on more than ERNs but also on many other stakeholders, such as policymakers, industry, and health care payers, and their experiences and expectations. Nevertheless, medical professionals do represent a distinct link between patients and payers. Therefore, the insights provided by this community are of high interest for adopting novel ML technologies in diagnosis and reducing the delay in diagnosis. Furthermore, ERNs represent pan-European hubs of research and knowledge. Thus, they enjoy a special status in the ecosystem of RDs taking on a leadership role [2–4, 20, 21].

### Expected benefits and risks of ML-based RD diagnosis

We found a distinctly positive attitude among ERN members towards the use of ML for the diagnosis of RDs, despite the large share of respondents who indicated a lack of knowledge of and experience with such tools. This trend was even more pronounced in the participants who already had some ML experience. As the use of ML becomes more common in medicine and healthcare, the role of trustworthiness of these tools must be discussed [15]. This is a recurrent problem reported by previous research [15, 16, 24], which hinders the successful integration of ML-based tools into existing healthcare workflows [25]. There is a need for ML training curricula targeting medical specialists. However, their focus should be on enhancing ML literacy among clinicians rather than building technical skills [26].

Our focus group indicated that medical specialists should not be held responsible for being ML experts. It is important, however, to promote early dialogue and collaboration between ML developers and members of ERNs. ERNs can inform the research and development of novel ML-based tools for diagnosing RDs. Together, healthcare professionals and ML developers can design tailored ML training across all medical specialties. This kind of much-needed synergy could be the critical starting point for building trust in and willingness to adopt ML-based tools.

Participants firmly expected that the RD field would largely benefit from the adoption of ML-based diagnostic tools. Rapid and reliable disease diagnosis, as well as the synthesis of clinical information, were deemed to be the most significant benefits of this new paradigm. ML-based RD diagnosis seems to be a promising technology, and the reported positive attitudes could be easily transformed into routine clinical application [27]. However, like other studies before, the diagnostic ability of ML is regarded as subject to a physician’s evaluation and final decision [28].

The latter aspect was repeatedly underlined by our focus group attendees. They stressed the fact that interpretation of the findings and communication with patients and their families represent integral parts of the RD diagnostic process and cannot be substituted by any technology. In fact, our participants considered ML-based self-diagnosis by patients very problematic with the current development stage of ML technologies. Contrarily to some previously reported results, reputational loss and reduced demand for specialist opinion due to ML implementation were perceived as the least important potential risks [24].

### Preferred implementation modalities of ML-based RD diagnosis

We explored the level of agreement about different implementation modalities of ML-based RD diagnosis. One topic for which our study revealed strongly diverging opinions among ERN members was the secondary use of anonymous ML-based diagnostic findings, which highlight the complex ethical considerations surrounding data sharing and consent in the context of ML technologies [29]. While most of our survey’s respondents believed that anonymized ML-generated diagnostic data should be available for secondary use only with patients’ consent (consent required), a substantial minority were open to the idea of sharing anonymized ML-generated diagnostic data for secondary research if patients did not explicitly opt-out. Further debating the issues of data privacy protection, previous studies have also underlined the importance of establishing guidelines and frameworks to ensure accountability and responsibility in cases of breaches and hacking [30–32].

Public funding was clearly preferred as the main source to support ML-based diagnostics of RDs in both our survey and focus group. However, this is not surprising, as we did not ask healthcare payers, such as health insurance companies or national health authorities. One of the participants explicitly tied the question of funding to the issue of equity and equal access to diagnosis and treatment. In the end, it will be important to distinguish between research endeavours and routine ML-based diagnosis. Our results indicate physicians’ preference for financing the latter via a mix of public and private resources.

Most of our study’s participants agreed that ML’s application in the next five years should be restricted to specialised units. We interpret the tendency to specialisation as ML-based technologies being perceived as having the most immediate impact and the biggest potential in tertiary clinical settings, where there is a substantial accumulation of expertise and resources for RDs [20].

However, even if ML technologies spread across healthcare settings, the respondents highlighted the importance of the patient-physician relationship and the interpretation of diagnostic findings, which cannot be replaced by technology. This impact of ML on the medical profession has been underlined by several studies [33]. In particular, ML tools for patient use offer a more optimistic outlook on this complex matter, although healthcare systems’ infrastructure might not be ready to facilitate autonomous ML usage by patients [34].

### Limitations

This study has a number of limitations. Convenience sampling was applied, thus some ERNs may be under- or overrepresented in the sample of responses. Therefore, our findings may not be considered fully representative of all ERNs’ attitudes and opinions about ML-based diagnostics of RDs. Nevertheless, to our best knowledge, this is the first attempt to explore the question of ML acceptance among ERNs. While we consider that our study marks an important starting point, the debate around routine application of ML for the diagnosis of RDs is expected to continue in the future.

Second, our survey respondents reported a limited degree of knowledge and experience with ML. This relative lack of expertise may have been reflected in the overall results and conclusions of our research. Nonetheless, we believe that this specific outcome well describes the current RD ecosystem in Europe regarding ERN members’ perception and understanding of this novel technology.

Third, our research provides information on ERNs’ members only. Professionals from other levels of the health care system, especially primary care specialists, should be consulted as well. Successful implementation of ML-based diagnostic tools for RDs, including coverage and reimbursement, will need to be agreed upon and worked on by various other stakeholders as well, including patient organisations. Therefore, it is equally important to also explore the attitudes and expectations of these specific groups.

Last but not least, our study did not explore the technical aspects of ML-based diagnostics for RDs. It is equally important that the findability, accessibility, interoperability, and reusability of the existing RD data sources are surveyed and analysed in order to provide a more objective overview of the short- and long-term prospects of the ML-based diagnosis of RDs in the EU. Within the Screen4Care project, there is a separate work task to address the latter question [35], and its research outcomes would greatly inform this ongoing debate.

## Conclusions

We found enthusiasm to implement and apply ML technologies, especially diagnostic tools in the field of RDs, despite the perceived lack of experience. While these findings are subject to limitations, to our best knowledge, they provide the first insights into that complex issue and could serve as a starting point for further research on the potential use of ML within the ERNs. Early dialogue and collaboration between health care professionals, developers, industry, policymakers, and patient associations seem to be crucial to building trust, improving performance, and ultimately increasing the willingness to accept diagnostics based on ML technologies.

## Data Availability

The data that support the findings of this study are not openly available due to reasons of sensitivity and are available from the corresponding author upon reasonable request. Data are located in controlled access data storage at the Institute for Rare Diseases (Plovdiv, Bulgaria).

## Appendix

See Tables 1 and 2.

**Table 1:** Survey questionnaire

Socio-demographic and career profile	
Gender:	Male Female Rather not say Other:
Age in years:	
Country:	Austria Belgium Bulgaria Croatia Republic of Cyprus Czech Republic Denmark Estonia Finland France Germany Greece Hungary Ireland Italy Latvia Lithuania Luxembourg Malta Netherlands Poland Portugal Romania Slovakia Slovenia Spain Sweden Other, please specify:
Affiliated European reference network (multiple responses allowed)	ERN BOND – European Reference Network on bone disorders ERN CRANIO – European Reference Network on craniofacial anomalies and ear, nose and throat (ENT) disorders Endo-ERN – European Reference Network on endocrine conditions ERN EpiCARE – European Reference Network on epilepsies ERKNet – European Reference Network on kidney diseases ERN-RND – European Reference Network on neurological diseases ERNICA – European Reference Network on inherited and congenital anomalies ERN LUNG – European Reference Network on respiratory diseases ERN Skin – European Reference Network on skin disorders ERN EURACAN – European Reference Network on adult cancers (solid tumours) ERN EuroBloodNet – European Reference Network on haematological diseases ERN eUROGEN – European Reference Network on urogenital diseases and conditions ERN EURO-NMD – European Reference Network on neuromuscular diseases ERN EYE – European Reference Network on eye diseases ERN GENTURIS – European Reference Network on genetic tumour risk syndromes ERN GUARD-HEART – European Reference Network on diseases of the heart ERN ITHACA – European Reference Network on congenital malformations and rare intellectual disability MetabERN – European Reference Network on hereditary metabolic disorders ERN PaedCan – European Reference Network on paediatric cancer (haemato-oncology) ERN RARE-LIVER – European Reference Network on hepatological diseases ERN ReCONNET – European Reference Network on connective tissue and musculoskeletal diseases ERN RITA – European Reference Network on immunodeficiency, autoinflammatory and autoimmune diseases ERN TRANSPLANT-CHILD – European Reference Network on Transplantation in Children VASCERN – European Reference Network on Rare Multisystemic Vascular Diseases
Medical specialty (multiple responses allowed)	Accident and emergency medicine Allergology Anaesthetics Biological hematology Cardiology Child psychiatry Clinical biology Clinical chemistry Clinical neurophysiology Clinical radiology Dental, oral and maxillo-facial surgery Dermatology Dermato-venerology Endocrinology Gastro-enterologic surgery Gastroenterology General hematology General practice General surgery Geriatrics Immunology Infectious diseases Internal medicine Laboratory medicine Maxillo-facial surgery Microbiology Nephrology Neurology Neuro-psychiatry Neurosurgery Nuclear medicine Obstetrics and gynecology Occupational medicine Ophthalmology Orthopaedics Otorhinolaryngology Paediatric surgery Paediatrics Pathology Pharmacology Physical medicine and rehabilitation Plastic surgery Podiatric medicine Podiatric surgery Psychiatry Public health and preventive medicine Radiology Radiotherapy Respiratory medicine Rheumatology Stomatology Thoracic surgery Tropical medicine Urology Vascular surgery Venereology Other, please specify:
Professional experience in years:	
Main professional sector (> 50% of the working time):	Public Private Equally
Main professional role:	Administration Diagnosis and treatment Research Teaching Other, please specify:
Participant’s knowledge and attitude towards machine learning	
How would you assess your knowledge of ML on a 1–5 scale (1 being the lowest and 5 being the highest)?	
Have you ever used ML in your clinical practice (for example, as a research project or as a routine technique)?	Yes, I have extensive experience Yes, I have limited experience No, I have no experience
Based on your knowledge on and experience with ML so far, how would you assess each one of the following potential benefits of ML on a 1–5 scale (1 being the least important and 5 being the most important)?	Improved diagnostic accuracy More efficient workflows Less time spent on administrative and other mundane tasks Synthesis of clinical information Updating of clinical records More time spent with patients Improved access to care
Would you like to comment on other potential benefits of ML that are not listed above?	
Based on your knowledge on and experience with ML so far, how would you assess each one of the following potential risks of ML on a 1–5 scale (1 being the least important and 5 being the most important)?	Liability for ML-mediated errors Insufficient training and continuing professional development in ML Reputational loss and reduced demand for specialist opinion Potential erosion of empathetic communication with patients Risk of privacy breaches and loss of confidentiality of patient information Lack of proof of efficacy of ML applications in clinical settings Lack of accuracy, fairness, transparency and decision-making power of the ML outcomes
Would you like to comment on other potential risks of ML that are not listed above?	
Participant’s attitudes towards machine learning’s potential implementation and integration in healthcare	
In case of ML being routinely applied in the diagnostic process, what do you think is the most appropriate way to mandate this process?	ML should be a mandatory part of the diagnostic process ML should be an optional, but recommended part of the diagnostic process ML should be available only upon patient’s request Other, please specify:
In case of ML being routinely applied in the diagnostic process, what types of ML results should be disclosed to patients?	All results should be disclosed to patients Scope and type of results to be disclosed should be regulated at EU level (for example, EU regulation) Scope and type of results to be disclosed should be regulated at national level (for example, national regulation) Scope and type of results to be disclosed should be regulated by local medical associations (for example, guidelines) Physicians should choose what types of results to disclose to patients Patients should choose what types of results they would like to receive Physicians should choose what types of results to disclose to patients with an option of further decision by the patient Other, please specify:
In case of ML being routinely applied in the diagnostic process, what do you think should be the main source to fund this activity?	Public funding (for example, government subsidy, reimbursement, etc.) Private funding (for example, direct payment by the patients) Mixed (public–private funding) Funding through research projects Funding through research projects and subsequent public funding if justified Other, please specify:
In case of ML being routinely applied in the diagnostic process, what do you think is the most appropriate way to regulate secondary use of anonymized ML-generated diagnostic data?	Anonymized ML-generated diagnostic data should not be available for secondary use Anonymized ML-generated diagnostic data should be available for secondary use only with patients’ consent (consent required) Anonymized ML-generated diagnostic data should be available for secondary use without patients’ consent, but patients can opt out (assumed consent) Anonymized ML-generated diagnostic data should be available for secondary use without patients’ consent (no consent required) Other, please specify:
Participant’s attitudes towards machine learning’s prospects	
What would you expect the application of ML to be in the next 5 years?	ML is routinely applied in all clinical settings and all levels of health care, including autonomously by patients themselves (wide application with no restrictions) ML is routinely applied only in designated centres of expertise and European reference networks (restricted application in specialized units only) ML is only applied within the framework of research projects (no change from the current situation)
How would you assess each one of the following influencing factors, so you could promote the routine application of and access to ML outside research projects? (1 being the least important and 5 being the most important)?	Ensuring accuracy, freedom from bias, trustworthiness Improving efficiency and reducing administrative burden Improving clinical decision-making and outcomes Maintaining the integrity of clinician – patient relationships Preserving professional status Obtaining regulatory approval Determining liability for error Ensuring data privacy, confidentiality and security Ensuring access and equity
How would you assess each one of the following criteria, so you could promote the routine application of and access to ML outside research projects? (1 being the least important and 5 being the most important)? ML diagnostic tools must be:	Based on models that have involved domain experts and have minimised bias Fitted to and complement routine clinical workflows and, where possible, self-populate the required data with minimal clinician input Shown to be as or more effective in improving clinical decision-making than current care Not distracting from, or degrading, human to human interaction and shared decision-making Developed and assessed with an eye to maximising explainability and transparency in regards to their inner workings Implemented with care regarding potential loss of jobs or professional reputation Subject to regulatory standards that are robust, transparent and responsive to updates of existing applications Associated with clear lines of responsibility regarding liability for error Adhering to legal and community expectations regarding privacy, confidentiality and security of health and medical data Equitably accessible to low income, remote or other disadvantaged populations
If you would like to comment on the survey and/or provide additional information and suggestions, please, use this field:	
Would you be willing to participate in an online focus group discussion with selected ERN stakeholders regarding the outcomes of this survey? If yes, please provide your name and contact e-mail in this field:

**Table 2:** Socio-demographic and career profile of the respondents

Characteristic	n (%)
*Country*	
Austria	10 (2.4)
Belgium	24 (5.7)
Bulgaria	4 (0.9)
Croatia	6 (1.4)
Czech Republic	9 (2.1)
Denmark	18 (4.3)
Estonia	8 (1.9)
Finland	6 (1.4)
France	28 (6.6)
Germany	38 (9)
Greece	8 (1.9)
Hungary	9 (2.1)
Ireland	3 (0.7)
Italy	96 (22.7)
Latvia	5 (1.2)
Lithuania	10 (2.4)
Luxembourg	1 (0.2)
Malta	2 (0.5)
Netherlands	38 (9)
Poland	13 (3.1)
Portugal	15 (3.5)
Republic of Cyprus	2 (0.5)
Romania	9 (2.1)
Slovakia	2 (0.5)
Slovenia	6 (1.4)
Spain	32 (7.6)
Sweden	13 (3.1)
Other	8 (1.9)
*Affiliated ERN (multiple responses allowed)*	
Endo-ERN – European Reference Network on endocrine conditions	72
ERKNet – European Reference Network on kidney diseases	129
ERN BOND – European Reference Network on bone disorders	12
ERN CRANIO – European Reference Network on craniofacial anomalies and ear, nose and throat disorders	12
ERN EpiCARE – European Reference Network on epilepsies	18
ERN EURACAN – European Reference Network on adult cancers (solid tumours)	21
ERN EURO-NMD – European Reference Network on neuromuscular diseases	6
ERN EuroBloodNet – European Reference Network on haematological diseases	19
ERN eUROGEN – European Reference Network on urogenital diseases and conditions	17
ERN EYE – European Reference Network on eye diseases	11
ERN GENTURIS – European Reference Network on genetic tumour risk syndromes	14
ERN GUARD-HEART – European Reference Network on diseases of the heart	10
ERN ITHACA – European Reference Network on congenital malformations and rare intellectual disability	22
ERN LUNG – European Reference Network on respiratory diseases	28
ERN PaedCan – European Reference Network on paediatric cancer (haemato-oncology)	13
ERN RARE-LIVER – European Reference Network on hepatological diseases	23
ERN ReCONNET – European Reference Network on connective tissue and musculoskeletal diseases	4
ERN RITA – European Reference Network on immunodeficiency, autoinflammatory and autoimmune diseases	10
ERN Skin – European Reference Network on skin disorders	12
ERN TRANSPLANT-CHILD – European Reference Network on Transplantation in Children	19
ERN-RND – European Reference Network on neurological diseases	12
ERNICA – European Reference Network on inherited and congenital anomalies	24
MetabERN – European Reference Network on hereditary metabolic disorders	26
VASCERN – European Reference Network on Rare Multisystemic Vascular Diseases	8
*Medical specialty (multiple responses allowed)*	
Accident and emergency medicine	1
Allergology	4
Anaesthetics	–
Biological hematology	2
Cardiology	18
Child psychiatry	1
Clinical biology	2
Clinical chemistry	–
Clinical neurophysiology	3
Clinical radiology	1
Dental, oral and maxillo-facial surgery	–
Dermatology	7
Dermato-venerology	9
Endocrinology	57
Gastro-enterologic surgery	2
Gastroenterology	15
General hematology	6
General practice	–
General surgery	3
Geriatrics	–
Immunology	9
Infectious diseases	–
Internal medicine	28
Laboratory medicine	5
Maxillo-facial surgery	2
Microbiology	–
Nephrology	102
Neurology	24
Neuro-psychiatry	4
Neurosurgery	5
Nuclear medicine	1
Obstetrics and gynecology	1
Occupational medicine	–
Ophthalmology	8
Orthopaedics	3
Otorhinolaryngology	3
Paediatric surgery	18
Paediatrics	128
Pathology	3
Pharmacology	–
Physical medicine and rehabilitation	1
Plastic surgery	2
Podiatric medicine	–
Podiatric surgery	2
Psychiatry	1
Public health and preventive medicine	2
Radiology	2
Radiotherapy	2
Respiratory medicine	21
Rheumatology	5
Stomatology	1
Thoracic surgery	–
Tropical medicine	–
Urology	5
Vascular surgery	–
Venereology	1
Other	13
*Main professional sector (> 50% of the working time)*	
Public	403 (95.3)
Private	11 (2.6)
Equally	9 (2.1)
*Main professional role*	
Administration	11 (2.6)
Diagnosis and treatment	353 (83.5)
Research	35 (8.3)
Teaching	9 (2.1)
Other	15 (3.5)

## Abbreviations

ERN: European Reference Network
ML: Machine learning
RD: Rare disease
